# Targeting the metabolic pathway of human colon cancer overcomes resistance to TRAIL-induced apoptosis

**DOI:** 10.1038/cddiscovery.2016.67

**Published:** 2016-09-12

**Authors:** Ryan M Carr, Guilin Qiao, Jianzhong Qin, Sundararajan Jayaraman, Bellur S Prabhakar, Ajay V Maker

**Affiliations:** 1 Department of Surgery, Division of Surgical Oncology, University of Illinois at Chicago, College of Medicine, Chicago, IL 60612, USA; 2 Department of Microbiology & Immunology, University of Illinois at Chicago, College of Medicine, 835S. Wolcott Ave., MC790, Chicago, IL 60612, USA; 3 The Creticos Cancer Center at Advocate Illinois Masonic Medical Center, Chicago, IL 60657, USA

## Abstract

Colon cancer is a leading cause of cancer-related mortality for which targeted therapy is needed; however, trials using apoptosis-inducing ligand monotherapy to overcome resistance to apoptosis have not shown clinical responses. Since colon cancer cells selectively uptake and rapidly metabolize glucose, a property utilized for clinical staging, we investigated mechanisms to alter glucose metabolism in order to selectively target the cancer cells and to overcome evasion of apoptosis. We demonstrate TRAIL (tumor necrosis factor-related apoptosis-inducing ligand) resistance in the majority of human colon cancers tested and utilize the glucose analog 2-deoxy-d-glucose to sensitize TRAIL-resistant gastrointestinal adenocarcinoma cells, and not normal gastrointestinal epithelial cells, to TRAIL-induced apoptosis through enhanced death receptor 5 expression, downstream modulation of MAPK signaling and subsequent miRNA expression modulation by increasing the expression of miR-494 via MEK activation. Further, established human colon cancer xenografts treated with this strategy experience anti-tumor responses. These findings in colon adenocarcinoma support further investigation of manipulation of cellular energetics to selectively overcome resistance to apoptosis and to impart tumor regressions in established colon cancer tumors.

## Introduction

Colorectal cancer is the third leading site of cancer in men and women and is the second leading cause of cancer-related deaths.^1,2^ Although the mortality of colorectal cancer has decreased by about 26% over the decades, only 3% has been due to improved treatment strategies.^3^ Chemotherapy is largely tumoristatic, has inherent morbidity that prohibits its use in patients with significant co-morbidities, and the best regimens provide on average less than 2 years of survival for patients with colorectal liver metastases. For these patients, new strategies and therapeutic approaches have been lacking and are profoundly needed. Our goal has been to identify strategies to selectively target colon cancer cells while leaving normal cells unperturbed.

The heightened metabolic demands of colon cancer cells result in increased glucose uptake and glycolytic flux relative to normal tissues. This property has been utilized to visualize colon cancer cells using positron emission tomography where signals emitted from 2-deoxy-2-fluoro-d-glucose (FDG), which is taken up preferentially by colon cancer cells, are quantified.^4^ Therefore, the established ability to discriminate between normal and malignant cells using a glucose analog suggests the possibility of using it as a tumor-specific therapeutic tool.

2-deoxy-d-glucose (2DG) is molecularly similar to FDG and is preferentially taken up by cancer cells. Its LD_50_ is well-established, and it has been safely used in clinical studies and multiple human clinical trials.^5–7^ In cancer cells, 2DG metabolism may affect death receptor (DR) expression and dissociate the Bak-Mcl-1 complex in cells with elevated glycolytic activity.^7^ Given its established safety, specific effect on only cancer cells, and ability to affect DR expression we were interested in evaluating DR ligands in combination with 2DG to mediate colon cancer-specific apoptosis.

Tumor necrosis factor-related apoptosis-inducing ligand (TRAIL) is a DR ligand that can induce apoptosis selectively in cancer cells with little-to-no effect on normal cells.^8^ TRAIL has been successfully utilized to suppress human tumor xenograft growth in several TRAIL-susceptible preclinical models;^9–12^ unfortunately, most human colon cancers are TRAIL resistant. Further, the use of recombinant human TRAIL in humans is safe and was well-tolerated in phase I and II clinical trials; however, objective clinical responses were rare and with no antitumor responses in patients with colon cancer.^8,13,14^ We and others have investigated mechanisms to overcome the known and highly prevalent causes of TRAIL resistance;^15,16^ however, the clinical trials highlighted the current limitations of TRAIL’s clinical use as a single agent.

Since 2DG has been shown to impact TRAIL’s cognate receptor expression (DR) *in vitro*, and both of these drugs are well characterized and proven safe in clinical trials, we sought to determine if 2DG treatment could sensitize TRAIL-resistant human colon cancers to TRAIL-induced apoptosis.

## Results

### 2DG sensitizes only cancer cells to TRAIL-induced apoptosis

**Figure 1:**
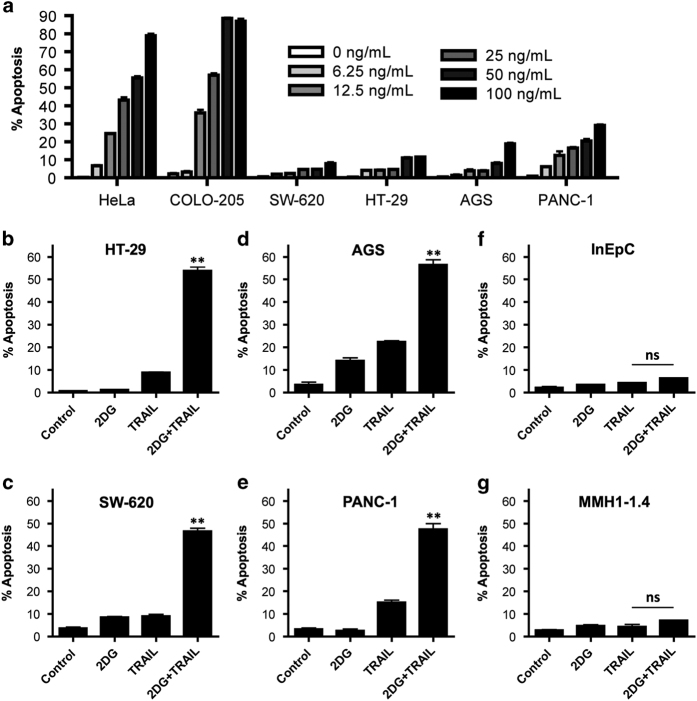
2DG synergizes with TRAIL to induce cell death in TRAIL-resistant cancer cell lines but not in normal cells. (**a**) HeLa cells (positive control) are sensitive to TRAIL-induced apoptosis; however, the majority of human colon (HT-29, SW-620), stomach (AGS) and pancreatic cancers (PANC-1) tested were TRAIL resistant. (**b**–**e**) TRAIL sensitization upon treatment with 2DG. (**f**, **g**) Nonmalignant cell controls (InEpC, normal human intestinal epithelial cells; MMH1-1.4, hepatocytes) show no effect of 2DG treatment on TRAIL-induced apoptosis. Apoptosis is detected by caspase-3 activation. *n*=3, and was validated with Annexin V+PI and TMRM staining (Supplementary Figure S1). ** indicates *P*<0.01, ns indicates *P*>0.05.

TRAIL dose–response experiments in human colorectal cancer cells (COLO-205, SW-620 and HT-29), human gastric adenocarcinoma (AGS) and human pancreatic cancer cells (PANC-1) were performed. HeLa cells, in which we have demonstrated TRAIL sensitivity,^17^ served as a positive control. All the malignant cell lines tested were highly resistant to TRAIL-induced apoptosis with the exception of COLO-205 (Figure 1a).

None of the TRAIL-resistant cell lines were susceptible to cell death induced by either 10 mM 2DG or 50 ng/ml TRAIL alone. However, upon administration of both compounds, dramatic synergy in cell death was observed (Figures 1b–e). Significant tumor cell apoptosis was identified in all TRAIL-resistant human cancer cell lines, including >6× cell death in the colon cancer cell line HT-29 (53.8%±2.8 *versus* 8.85%±0.4, *P*=0.001).

We investigated whether 2DG+TRAIL would cause toxicity or cell death in normal cells. As our interest is primarily in gastrointestinal/colon adenocarcinomas that grow in the luminal epithelium of the gastrointestinal tract and most often metastasize to the liver, non-cancerous intestinal epithelial cells and hepatocytes were chosen. When treating InEpC (normal intestinal epithelial cells; Figure 1f) and MMH1-1.4 (immortalized, non-transformed hepatocytes; Figure 1g) with 2DG+TRAIL, no increase in cell death or toxicity was identified.

### 2DG and TRAIL propagate apoptosis through the extrinsic pathway and cleave Bid

**Figure 2:**
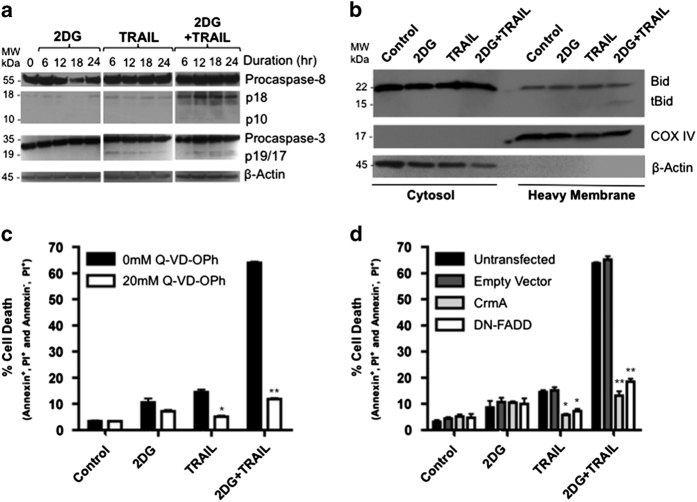
2DG+ TRAIL enhance activation and are dependent upon the extrinsic apoptotic pathway to induce colon cancer cell death. (**a**) Treating TRAIL-resistant HT29 colon cancer cells with 2DG, TRAIL or 2DG+TRAIL resulted in robust caspase-8 and caspase-3 activation with combination therapy. Cleavage products p18 and p10 are indicative of caspase-8 activation while p19 and p17 represent caspase-3 activation. (**b**) HT-29 cells were fractionated into cytoplasmic and membrane heavy fractions. Truncated Bid (tBid) in the membrane heavy fraction was only found in the 2DG+TRAIL-treated cells. (**c**) HT-29 cells were treated with the general caspase inhibitor, Q-VD-OPh. Apoptosis was assessed by Annexin V/PI staining revealing inhibition of 2DG+TRAIL-induced apoptosis. (**d**) HT-29 cells were stably transfected with CrmA, DN-FADD plasmid constructs or an empty vector. Blockade of the extrinsic apoptotic pathway abrogated 2DG and TRAIL synergy. *n*=3, * indicates *P*<0.05, ** indicates *P*<0.01.

TRAIL induces apoptosis through the sequential activation of caspase-8 and caspase-3. 2DG treatment had no effect on the activation of either caspase-3 or -8 and TRAIL alone demonstrated minimal activation of these caspases in HT29 cells. The combination of 2DG and TRAIL resulted in robust activation of both caspase-3 and -8 as assessed by accumulation of their cleavage products p18/p10 and p19/p17, respectively (Figure 2a). Further, activated caspase-8 is able to directly cleave Bid in type II HT-29 cells, resulting in its translocation to the mitochondria; therefore, we evaluated the effect of combination treatment on Bid cleavage. Neither 2DG nor TRAIL treatment resulted in Bid translocation; however, 2DG+TRAIL treatment elevated levels of truncated Bid (tBid) (Figure 2b). Collectively, these data support that in these TRAIL-resistant colon cancer cells, 2DG+TRAIL treatment induced apoptosis through the extrinsic pathway.

### 2DG+TRAIL-induced apoptosis is dependent on the extrinsic pathway

To determine if the apoptosis resulting from 2DG+TRAIL treatment was dependent on initiation of the extrinsic apoptotic pathway, cells were treated with or without 20 *μ*M of the general caspase inhibitor, Q-VD-OPh. The general caspase inhibitor completely abrogated the synergy between 2DG and TRAIL-induced apoptosis to levels comparable to TRAIL or 2DG treatment alone (Figure 2c).

To more specifically determine that the effect was propagated through the death-inducing signaling complex (DISC) and caspase-8 activation, we generated HT-29 cell lines stably transfected with either CrmA, a serpin that specifically inhibits caspase-8, or dominant-negative FADD (DN-FADD). Similar to Q-VD-OPh, expression of CrmA or DN-FADD both significantly abrogated cell death in 2DG+TRAIL-treated cells (Figure 2d). Taken together, these data demonstrate a dependence on the extrinsic apoptotic pathway for 2DG+TRAIL synergy.

### 2DG increases mRNA, total protein levels and surface expression of DR5 but not DR4

**Figure 3:**
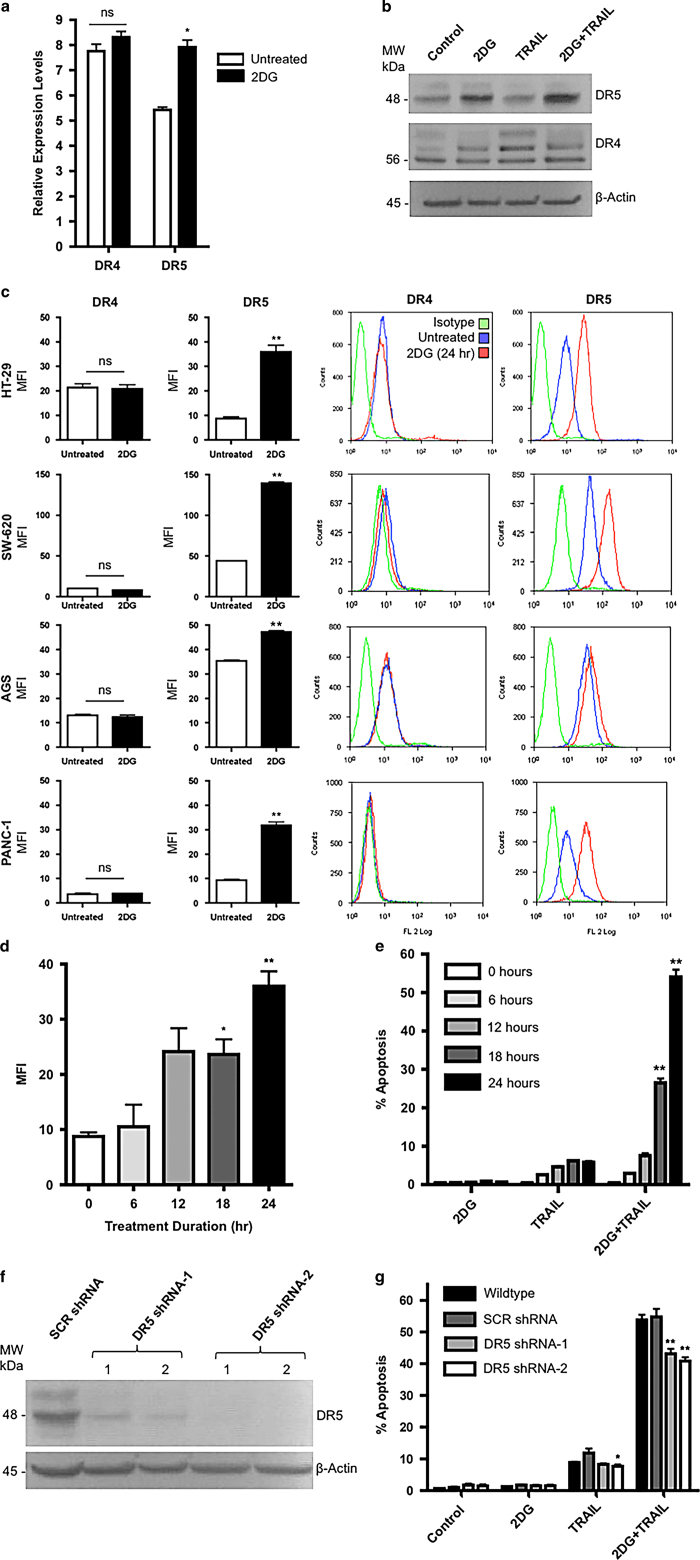
2DG increases DR5 expression levels with no effect on DR4, however, the increase in TRAIL-induced apoptosis requires additional mechanisms. (**a**) QRT-PCR of DR4 and DR5 transcripts in HT29 cells with and without 2DG treatment. (**b**) Immunoblotting for DR4 and DR5 indicated increased DR5 protein expression with 2DG treatment. (**c**) DR surface expression in various TRAIL resistant colon, gastric and pancreatic adenocarcinoma cells. (**d**) DR5 surface expression increases with 2DG treatment over time in HT-29 cells. (**e**) Apoptosis, as assessed by capsase-3 activation, in the same group of HT-29 cells increased over a 24 h time course upon treatment with 2DG+TRAIL, corresponding temporally with the increase in DR5 expression. (**f**) To knock down DR5 expression, HT-29 cells were transduced with the indicated lentiviral constructs and allowed to grow for 48 h. A lentivirus expressing a scrambled sequence was used as a control. (**g**) Cell death was significantly decreased with DR5 knockdown in 2DG+TRAIL-treated cells, however, remained significantly higher than wild-type cells treated with 2DG or TRAIL alone. *n*=3, ** indicates *P*<0.01, ns indicates *P*>0.05.

Relative to untreated controls, 2DG treatment had no significant effect on steady-state transcript levels of DR4; however, mRNA levels of DR5 were significantly increased (Figure 3a). Similarly, 2DG treatment increased protein levels of DR5 but not DR4. TRAIL treatment had no effect on DR5 protein expression (Figure 3b). When evaluated by flow cytometry, the results were corroboratory with DR4 surface expression not significantly changed upon 2DG treatment and DR5 levels significantly increased by 4.1-, 3.1-, 1.3- and 3.4-fold in HT-29, SW-620, AGS and PANC-1 cells, respectively (Figure 3c). Therefore, DR5 transcript, protein and surface expression were increased upon 2DG treatment.

### DR5 surface expression correlates with TRAIL sensitivity but knockdown does not completely abrogate the synergy between 2DG and TRAIL

Treating HT-29 cells with 2DG resulted in a step-wise and significant increase in DR5 surface expression over 24 h (Figure 3d). This correlated with increased cell death over the same time course with 2DG+TRAIL (Figure 3e). To determine if 2DG-induced overexpression of DR5 was necessary for 2DG+TRAIL synergy we designed two lentiviral constructs to deliver shRNA targeted against DR5. Within 48 h of transduction, both shRNA clones were able to efficiently knock down DR5 protein expression in HT-29 cells relative to cells transduced with a scrambled (SCR) control (Figure 3f). Cell death with 2DG+TRAIL was attenuated by approximately 25% in the absence of DR5, but remained significantly greater than when treated with TRAIL alone (Figure 3g). These data, in combination with the minimal increase in surface DR5 expression in the 2DG-treated AGS cell line, suggest that DR5-mediated apoptosis accounts for some, but not all, of the effect of 2DG+TRAIL-induced apoptosis.

### 2DG and TRAIL treatment altered microRNA 494 levels in human colon cancer cells

Since 2DG-stimulated upregulation of DR5 expression was not solely responsible for the enhanced susceptibility to 2DG+TRAIL treatment, we were interested in evaluating other mediators affecting the increased sensitivity to 2DG+TRAIL-induced apoptosis. As microRNAs (miRNAs or miRs) have been implicated as regulators of apoptosis and TRAIL-induced cell death, the effect of 2DG on miRs was further investigated. HT-29 cells were untreated or cultured for 18 h with 2DG, TRAIL or 2DG+TRAIL. Of 800 miRNAs evaluated, 42 miRNAs were significantly up- or downregulated (Supplementary Data and Supplementary Table S1). The ratio of miRNA expression after 2DG+TRAIL treatment to treatment with TRAIL or 2DG alone identified biologically relevant candidate miRNAs with at least a twofold change in expression attributable to 2DG+TRAIL synergy.

**Figure 4:**
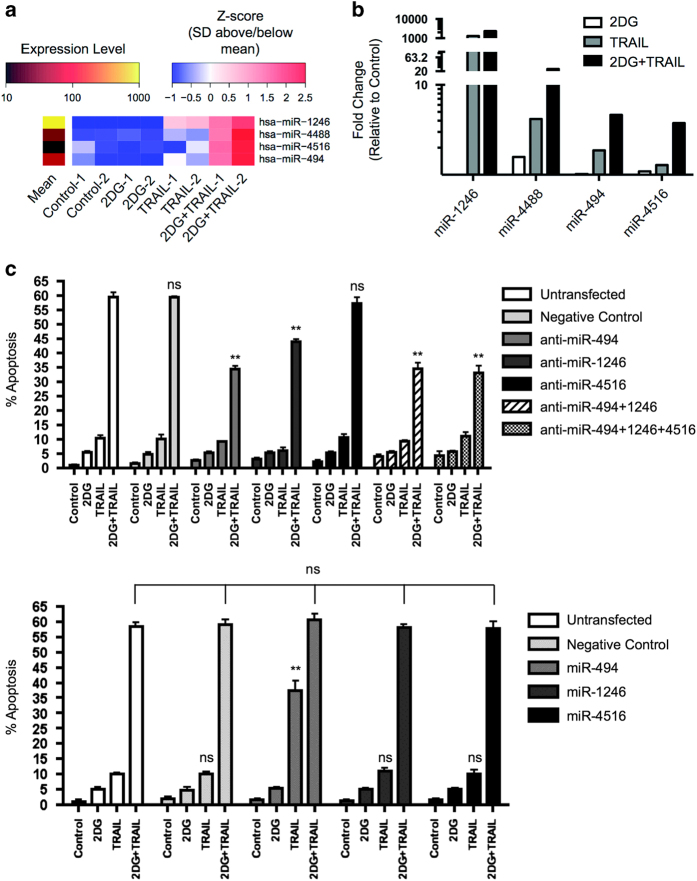
MicroRNA levels are increased with 2DG+TRAIL treatment and the effect of miR inhibitors and mimics on 2DG+TRAIL-induced apoptosis. (**a**) Heat map demonstrating relative increased expression levels of miR-1246, -4488, -4516 and -494 with combination treatment (shown in biological duplicates from two separate experiments). MiR-expression is upregulated and clusters in the 2DG+TRAIL-treated cells. (**b**) Fold change of miRs relative to untreated controls reveal increased miR-1246, -4488, -494 and -4516. Experiment was repeated in duplicate with Nanostring nCounter gene expression analysis and expression patterns were validated with RT-PCR. (**c**) HT-29 cells were transfected with miR inhibitors (upper panel) or mimics (lower panel). Untransfected cells and those transfected with a negative control were used as controls. Twenty-four hours post-transfection, cells were treated with 2DG, TRAIL or 2DG+TRAIL for 24 h. Inhibited expression of miRs 494 and 1246 significantly abrogated the effect of 2DG+TRAIL on apoptosis. Only overexpression of miR-494 (lower panel) sensitized cells to TRAIL-induced apoptosis even without the addition of 2DG. Apoptosis was assessed by measuring caspase-3 activation. *n*=3, ** indicates *P*<0.01, * indicates *P*<0.05, ns indicates *P*>0.05.

MiR-494, miR-4488 and miR-4516 were upregulated by 2.5-, 5.7- and 2.9-fold, respectively, under conditions of 2DG+TRAIL treatment compared with TRAIL alone, and 4.5-, 15.0- and 3.4-fold compared with 2DG alone. MiR-1246 was also included in the analysis given that it was the most highly expressed miRNA with the combination treatment at 2313-fold over control (2DG+TRAIL:TRAIL ratio +1.8, 2DG+TRAIL:2DG ratio +2891) (Figure 4). qRT-PCR was performed to validate the NanoString analysis with transcript evaluation. miR-4488 levels were very low and not detected by qRT-PCR and miR-494, miR-1246 and miR-4516 were significantly upregulated in cells treated with 2DG+TRAIL relative to controls treated with TRAIL or 2DG alone, validating the array data.

### miR-494 expression sensitizes cancer cells to TRAIL-induced apoptosis

To determine if the change in phenotype with 2DG sensitization was secondary to the effect of these miRNAs on post-transcriptional modification, the target miRNAs were under- or over-expressed and apoptosis determined with various treatments (Figure 4). HT-29 cells were electroporated with individual microRNA inhibitors and mimics for miR-494, miR-1246 or miR-4561. qRT-PCR for each microRNA was performed to confirm the efficacy of knockdown or overexpression (Supplementary Figure S2). Knockdown of miR-4516 in the colon cancer cells did not abrogate tumor cell apoptosis upon treatment with 2DG+TRAIL. However, knockdown of either or both miR-494 and -1246 significantly decreased, but did not abolish, the apoptotic effect of 2DG+TRAIL. To assess whether the miRNA may be functioning synergistically, we simultaneously knocked down a combination of both 494 and 1246, or 494, 1246 and 4516. The combined antagonism of miRNAs did not decrease apoptosis to a greater extent than knockdown of 494 alone, indicating the singular importance of miR-494 in enhancing 2DG+TRAIL-induced apoptosis (Figure 4).

Elevated expression of miR-1246 and -4516 had no effect on apoptosis; however, overexpression of only miR-494 conferred a significant increase in sensitivity to TRAIL (Figure 4, lower panel). This sensitivity was not enhanced further when combining the miR-494 mimic with those of miR-1246 and miR-4516. Therefore, decreased miR-494 expression appeared to be associated with compromised apoptosis in 2DG+TRAIL-treated cells, and increased miR-494 expression was associated with conferring sensitization to TRAIL-induced apoptosis. 2DG alone did not directly enhance miR-494 expression; therefore, these data suggest that 2DG treatment enables enhanced miR-494 expression in the context of TRAIL treatment which enables apoptosis.

### MEK signaling is necessary for 2DG+TRAIL synergy in inducing apoptosis in colon cancer cells

**Figure 5:**
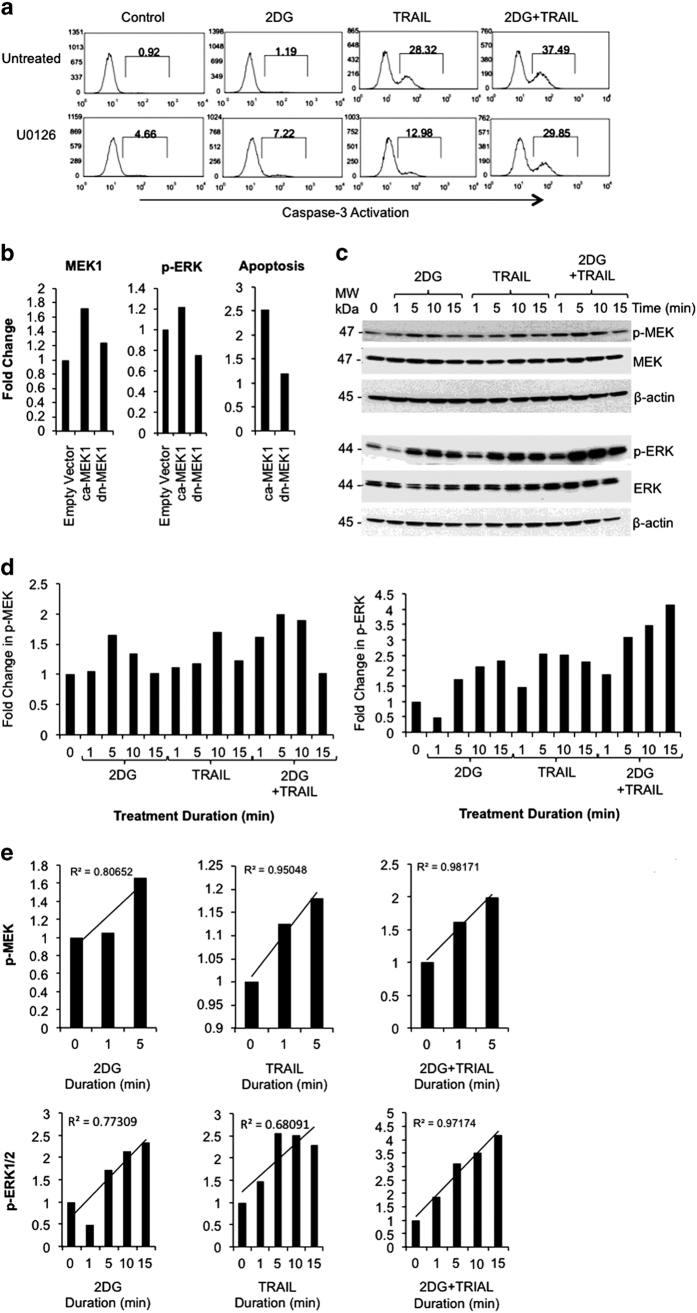
MEK signaling is necessary for 2DG+TRAIL synergy in inducing apoptosis. (**a**) MEK inhibition decreased apoptosis in 2DG+TRAIL-treated cells to levels comparable to TRAIL alone. (**b**) Cells were engineered to express constitutively activated (ca) MEK1 (1.8× compared with empty vector) or dominant-negative (dn) MEK1 (1.2× compared with empty vector). Protein expression on western blot analysis demonstrated an expected increase or decrease in phospho(p)-ERK1/2 (1.4× *versus* 0.8×, respectively) resulting in increased or decreased apoptosis with caMEK1 and dnMEK1, respectively. Levels of activated caspase-3 after 24 h in cells with CA-MEK were increased 2.5-fold (6.7% *versus* 2.65%) over control cells transfected with an empty vector, and 2.1-fold (6.7% *versus* 3.2%) over DN-MEK cells (**c**) Western blot analysis of protein expression of p-MEK and ERK was (**d**) adjusted for b-actin expression. (**e**) pMEK levels reached peak expression faster (increased slope) and to a greater degree in cells treated with 2DG+TRAIL. The slope/peak of phosphorylation over this time period for 2DG-treated, TRAIL-treated and combination-treated cells was 0.33/1.66×, 0.09/1.18× and 0.5/1.99×. Similarly, p-ERK levels increased rapidly and to a greater degree in the 2DG+TRAIL-treated cells. The slope/peak of phosphorylation for 2DG-treated, TRAIL-treated and combination-treated cells was 0.43/2.34×, 0.36/2.56× and 0.79/4.15×.

We sought to determine critical regulators of miR-494. A screening assay using inhibitors of kinases involved in MAP kinase signaling was performed. The MAPK signaling inhibitors PD98059, U-0126 and SB-20358 abrogated apoptosis in the 2DG+TRAIL-treated group at all concentrations tested to levels comparable with untreated controls and had no effect on the untreated, 2DG or TRAIL alone treated groups (Supplementary Table S2). In particular, U-0126, an inhibitor of MEK1/2, demonstrated increasing amounts of apoptosis inhibition with increased drug dose. Therefore, we chose U-0126 for validation and treated HT-29 cells with U-0126 *in vitro* and evaluated for caspase-3 activation by flow cytometry (Figure 5a). Apoptosis in 2DG+TRAIL-treated cancer cells was inhibited to levels comparable to treatment with TRAIL alone. The experiment was repeated three times confirming significant reduction in apoptosis in the 2DG+TRAIL group upon treatment with the MEK inhibitor (*P*=0.04) and that the level of apoptosis in this treated group was comparable to treatment with TRAIL alone (*P*=0.44; Figure 5a).

To validate the importance of MEK signaling in mediating the synergy of 2DG+TRAIL in enhancing apoptosis in HT-29 cells we transfected the cells with constitutively activated (CA) MEK1 or dominant-negative (DN) MEK1. Increased transcript levels of MEK1 were confirmed, as was the functionality of CA-MEK, by demonstrating corresponding increases and decreases in p-ERK transcript levels with CA and DN-MEK expression, respectively. Levels of activated caspase-3 in cells with CA-MEK were increased 2.5-fold over control cells transfected with an empty vector, and 2.1-fold over DN-MEK cells (Figure 5b).

Levels of MEK, ERK, and phospho-MEK and phospho-ERK were then determined after treatment with 2DG, TRAIL or 2DG+TRAIL. Compared with 2DG or TRAIL treatment alone, p-MEK levels reached peak expression faster and to a greater degree in cells treated with 2DG+TRAIL. Similarly, p-ERK levels increased rapidly and to a greater degree in the 2DG+TRAIL-treated cells, and expression continued to rise after p-MEK levels started to return to normal (Figures 5c–e). At 2 h, p-ERK expression was maintained in 2DG+TRAIL-treated cells.

### MEK1/2 is upstream of miR-494 and regulates its expression in colon cancer cells treated with 2DG+TRAIL

**Figure 6:**
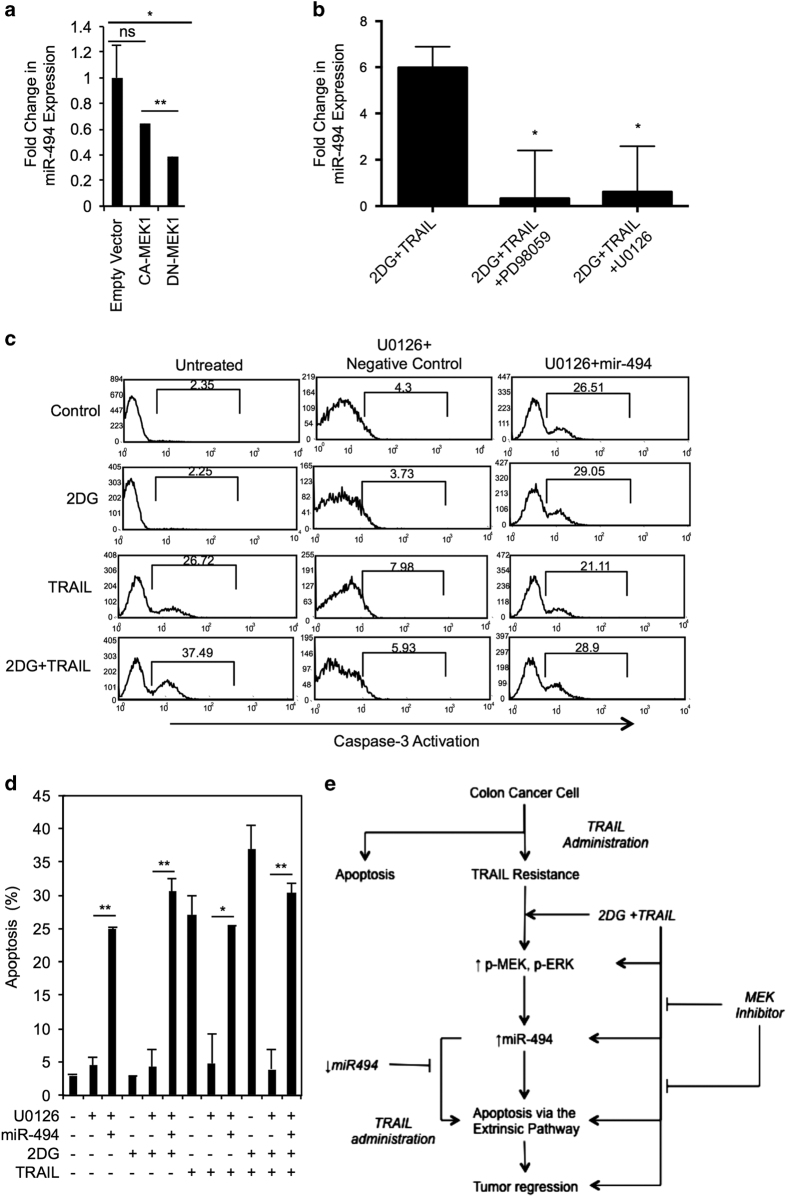
MEK1/2 are upstream of miR-494 and regulate its expression in colon cancer cells treated with 2DG+TRAIL. (**a**) miR-494 transcript levels were significantly increased in CA-MEK1-expressing cells compared with DN-MEK1 (**P=0.0002). (**b**) MEK inhibition with an MEK1 (PD98059) and an MEK1/2 inhibitor (U0126) caused miR-494 transcript levels in the 2DG+TRAIL-treated group to decrease 18- and 10-fold to near undetectable levels. (**c**, **d**) Caspase-3 activation was markedly enhanced with the combination treatment but was reduced to levels comparable to non-treated control cells in the presence of an MEK inhibitor. This inhibition of apoptosis was abrogated when these same cells were made to overexpress miR-494. *n*=3. (**e**) Summary diagram of the proposed pathway.

We sought to elucidate the relationship between miR-494 and MEK signaling. HT-29 cells were transfected with CA-MEK1 or DN-MEK1. Though not different than those cells transfected with empty vector, miR-494 transcript levels were significantly increased by 39% in CA-MEK1-expressing cells compared with DN-MEK cells (Figure 6a).

Next, colon cancer cells were co-incubated with PD98059 and U0126. With MEK inhibition, miR-494 transcript levels in the 2DG+TRAIL-treated group decreased 18- and 10-fold, respectively, to near undetectable levels (*P*<0.05; Figure 6b).

In the absence of U0126, caspase-3 activation was markedly enhanced with the combination treatment but was reduced to levels comparable to non-treated control cells in the presence of MEK inhibitor. This inhibition of apoptosis was reversed when these same cells were made to overexpress only miR-494 (Figures 6c and d). Therefore, miR-494 overexpression could rescue the apoptotic phenotype of 2DG+TRAIL treatment even in the presence of an MEK inhibitor. Together, these results imply that MEK expression affects, and is upstream of miR-494. The pathway is illustrated in Figure 6e.

### 2DG+TRAIL causes regression of established colon cancer tumors *in vivo*

**Figure 7:**
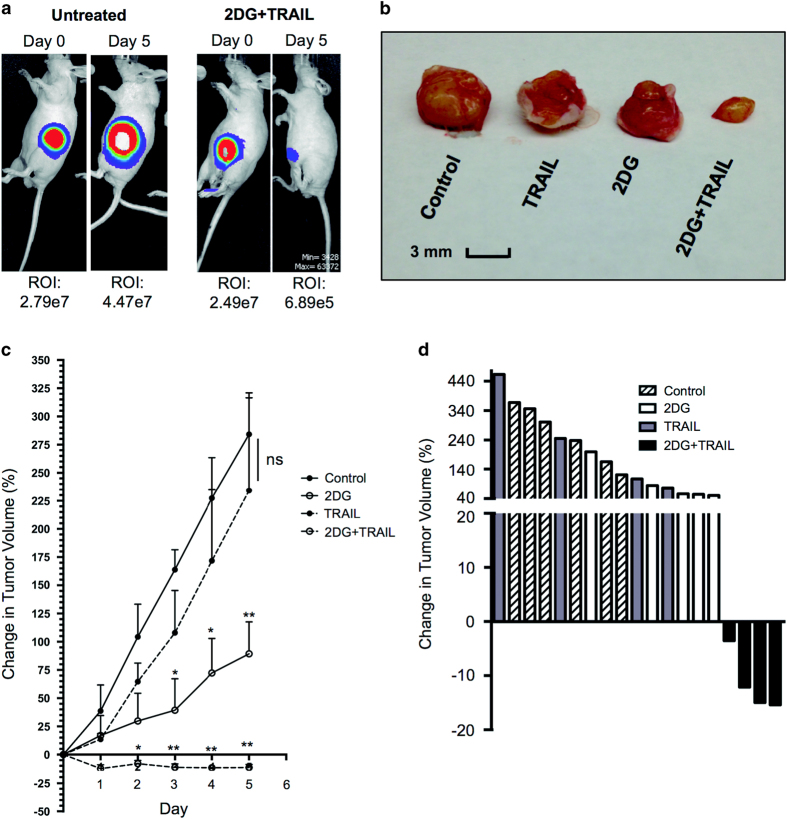
2DG and TRAIL cause regression of established solid tumors *in vivo*. (**a**) Representative images comparing tumor burden of an untreated mouse and one given 2DG+TRAIL before (day 0) and after (day 5) treatment following establishment of palpable tumors. (**b**) Photograph of representative tumors excised from mice of each experimental group after completion of treatment. (**c**) Tumor growth curves after initiation of treatment regimen. Data represent the percent change in tumor volume. (**d**) Waterfall plot where each bar represents a single mouse of the indicated treatment group. The data represent the percent change in tumor volume after 5 days of treatment relative to tumor volume on day 0. ** indicates *P*<0.01, * indicates *P*<0.05, ns indicates *P*>0.05.

The effect of this drug combination on established tumors *in vivo* remained to be determined and was critical to the translational applicability and pertinence of the study. Therefore, human colorectal cancer tumors were established in athymic nude mice. For clinical relevance, treatment was not initiated until there was an established, measurable and palpable tumor. Untreated mice experienced tumor growth of 3–4-fold over the treatment period. Mice treated with TRAIL alone demonstrated an aggressive increase in tumor volume not different from the tumor burden in the sham-treated control group. 2DG treatment alone attenuated the rate of tumor growth relative to both control and TRAIL-treated mice; however, tumors continued to grow and nearly doubled in volume. Animals treated with 2DG+TRAIL experienced significant tumor regression with clinical responses of up to 15% decrease in tumor volume over the treatment period (Figure 7). Control, TRAIL-treated and 2DG-treated tumors increased at 56.8, 46.8 and 17.8% per day. However, only 2DG+TRAIL treatment was able to decrease tumor burden, with a mean change in tumor volume of −2.3% per day. Animals in the combined treatment group maintained similar weight to control-treated animals and on autopsy no abnormalities or gross solid organ abnormalities were identified.

## Discussion

Previous research has identified TRAIL as a cytotoxic cancer cell-specific ligand with little to no effect on normal cells. Unfortunately, its clinical utility has been limited due to multiple mechanisms of TRAIL resistance.^18,19^ Colon cancer is a leading cause of cancer-related mortality for which targeted therapy is critically needed; however, clinical trials using TRAIL monotherapy have shown no response in these patients, and we similarly have demonstrated TRAIL resistance in the majority of human colon cancer cell lines tested. There is a gap in knowledge of how to sensitize resistant cells to the cytotoxic effects of TRAIL-induced apoptosis in gastrointestinal adenocarcinomas, and specifically colorectal cancer. We define a strategy to selectively target colon cancer cells by exploiting their propensity for increased glycolytic flux, using a non-metabolizable glucose analog to enhance sensitivity to TRAIL-induced apoptosis through enhanced DR5 expression combined with downstream modulation of MAPK signaling and subsequent miRNA expression modulation.

The glucose analog 2DG sensitized multiple TRAIL-resistant gastrointestinal adenocarcinoma cells, and importantly not normal gastrointestinal epithelial cells, to TRAIL-induced apoptosis. The inability of 2DG and TRAIL to induce apoptosis in normal intestinal epithelium and hepatocytes was encouraging for clinical applicability, especially considering that combinations of TRAIL with some chemotherapies have previously rendered hepatocytes sensitive to apoptosis.^20,21^ Further, complete abrogation of TRAIL sensitivity through blockade of DISC formation suggests that the enhanced extrinsic pathway is critical for 2DG and TRAIL synergy, confirming the mechanism of apoptosis.

That 2DG could significantly affect colon cancer tumor cell sensitivity to TRAIL led us to examine the mechanism of action. We hypothesized that the combination of 2DG and TRAIL would be highly specific for cancer cells not only because of the preferential uptake of 2DG, but also because cancer cells tend to express much higher levels of DR4 and DR5 relative to normal cells.^22^ Upregulation of DR5 surface expression with 2DG treatment was found in all cell lines tested; however, abrogation of DR5 expression was not sufficient to eliminate the synergy between 2DG and TRAIL, suggesting its upregulation was not the only determinant of TRAIL sensitivity conferred by 2DG treatment. Tumor cell surface levels of DR, furthermore, do not necessarily correlate with sensitivity to the death signal.^23^ Since we showed that 2DG treatment did not regulate DR4 levels, enhanced TRAIL sensitivity in the absence of DR5 suggested that 2DG must have other effects downstream of DRs in these cancer cells.

We therefore investigated downstream modulators of DR signaling and demonstrated that MEK and ERK activation were rapidly stimulated by 2DG+TRAIL. TRAIL treatment results in increased phosphorylation of MEK, and inhibition of MEK signaling can confer TRAIL resistance.^24^ The pathway herein elucidated implies that 2DG+TRAIL together can stimulate MEK signaling even in TRAIL-resistant cells. We further determined that 2DG treatment increased the duration and intensity of MEK activation in contrast to the transient activation of MEK after typical growth factor stimulation, and that this activation then sustained ERK activation for many hours. This was a critical finding since the longevity and intensity of the signal is what is critical to sustain the physiologic effect of this pathway.^25^ Normally, aberrant overexpression of MEK promotes proliferation and pro-tumorigenic signaling; however, interestingly in TRAIL-resistant cells, 2DG+TRAIL treatment appeared to phosphorylate MEK and subvert the pro-survival pathway through downstream signaling.

Activation of the MEK/ERK pathway is a major promoter of tumor development and is responsible for the transcription of many critical miRNAs.^26,27^ As miRNAs have been implicated as regulators of apoptosis and TRAIL-induced cell death,^28^ the effect of 2DG on post-transcriptional regulation was further investigated. 2DG+TRAIL treatment increased miR-494 levels, and increased miR-494 expression sensitized resistant cells to TRAIL-induced apoptosis. Constitutive activation of MEK increased miR-494, and inhibition of MEK, both by transfection with a dominant-negative MEK protein and with an MEK inhibitor, decreased miR-494 levels. This finding is consistent with miR-494 upregulation by ERK activation in non-malignant 293A cells.^26^ These observations highlight the increasing consensus that the physiologic relevance of each microRNA is highly contextual and cell type-dependent, and it critically demonstrates the complexity of miR transcription in various cancer types, for example, miR 494 may decrease in non-small cell lung cancer cells with ERK inactivation while enhancing TRAIL resistance. There is increasing consensus that the physiologic relevance of each microRNA is highly contextual and cell type-dependent, and that miR transcription in various cancer types may differ. Further, we have shown that 2DG+TRAIL-induced miR-494 overexpression can be abrogated with MEK inhibition and that forced overexpression of miR-494 in the presence of MEK inhibition rescues a pro-apoptotic phenotype, thereby establishing the importance of MEK signaling in the regulation of this miRNA’s expression and its upstream location in the signaling cascade. 2DG alone did not appear to directly enhance miR-494 expression but did enable enhanced miR-494 transcription upon TRAIL binding. Therefore, it follows that 2DG+TRAIL-induced colon cancer cell death by increasing the expression of miR-494 via MEK activation.

Finally, we demonstrated translational potential in established human colon cancer xenografts treated with the combination of 2DG+TRAIL to impart an anti-tumor response, consistent with *in vitro* experiments. These findings support the impact that this therapy, and that the elucidated mechanism, may have on the treatment of our patients with colon cancer and on the other TRAIL-resistant solid organ gastrointestinal malignancies that we tested, especially since the safety profile and lack of toxicity of both 2DG and TRAIL have been established in multiple human trials.^6,8,13,14,29,30^

Molecules not directly implicated in the TRAIL pathway are important to TRAIL receptor expression and may play a role in the 2DG+TRAIL pathway providing alternate explanations for the results, and warrant further investigation. Additionally, TRAIL receptor expression and the sensitivity of cells to TRAIL may be further regulated by other factors, including parallel signaling pathways and the complex cellular regulatory functions driven by cell-specific oncogenic mutations. Furthermore, glucose deprivation and 2DG administration may also affect sensitivity to apoptotic agents in a cell and tumor-dependent fashion.^31–33^ This includes sensitization to aoptosis by AMPK-mTOR control of MCL-1 and Bcl-2 family proteins, which can be either pro or anti-apoptotic depending on the cell type and inherent sensitivity to TRAIL. Therefore, alternative pathways of TRAIL resistance are likely present in non-gastrointestinal cancers, in cancers other than adenocarcinoma, or in different organs.^26^ Despite this limitation, we did evaluate the phenotype in additional colon, gastric and pancreatic cancer cell lines and observed similar results in apoptosis and DR expression. Our findings provide compelling data that miR-494 expression, when upregulated by 2DG+TRAIL treatment in colon cancer cells, leads to colon cancer cell TRAIL sensitivity. That this effect could be abrogated with an MEK inhibitor or Oncomir to miR-494 support our conclusions. In spite of the heterogeneity of mutations that result in multiple cancer types, and the consequent diversity of the underlying mechanisms by which 2DG and TRAIL could interact to induce apoptosis, this combination therapy was successful in multiple gastrointestinal cancer cell lines and did not affect normal cells. Extension of these data towards known mutations in different signaling pathways may establish additional potential targets for clinical utility in this most common of cancers that currently has poor survival and limited therapies in advanced disease.

## Materials and Methods

### Chemical reagents

Recombinant human Apo2L/TRAIL (PeproTech, Rocky Hill, NJ, USA (310-04)) was utilized as we have previously reported.^15,16^ 2DG (Sigma, St Louis, MO, USA (D8375)) dose was established at molar ratios from 0 : 1 to 25 : 1 with no difference in apoptosis of cancer cells across the spectrum. In DMEM that contained 2 g/l glucose, 10 mM 2DG was used consistent with previous studies and doses used safely in human clinical trials.^7^

### Tissue culture

HeLa, COLO-205, HT-29, SW-620, AGS and PANC-1 cancer cell lines were obtained from American Type Culture Collection (ATCC, Manassas, VA, USA) where each lot was STR profiled. HT-29 cells were transduced with the bicistronic lentiviral vector (pHIV1SDm-CMV-GFP-P2A-luc) (kindly provided by Dr Jeff Holst, University of Sydney, Sydney, Australia). Using a calcium precipitation method, a stably transduced cell line that expressed both GFP and luciferase was established. HeLa, HT-29 and PANC-1 cells were cultured in high glucose Dulbecco’s modified Eagle’s medium (Corning Cellgro, Tewksbury, MA, USA; 10-017-CV) without sodium pyruvate. COLO-205 cells were cultured in RPMI 1640 (Corning Cellgro; 10-040-CV). AGS cells were cultured in F-12K (ATCC; 30-2004) and SW-620 cells in L15 (ATCC; 30-2008). All media was supplemented with 10% fetal bovine serum (FBS) and 100 *μ*g/ml streptomycin.

Normal human intestinal epithelial cells (Lonza, Walkersville, MD, USA; 24972) were cultured according to the manufacturer’s protocol. Briefly, these cells were incubated in Smooth Muscle Cell Basal Medium (Lonza; CC-3181). Media was supplemented with smooth muscle cell medium BulletKit (Lonza; CC-3182) containing 0.5 ml insulin, 1 ml hFGF-B, 0.5 ml hEGF, 25 ml FBS and 0.5 ml GA-1000. Cells were cultured in dishes coated with rat tail collagen type I (BD Biosciences, San Jose, CA, USA; 354236). Cells were cultured at 33 °C with 5% CO_2_. MMH1-1.4 immortalized hepatocytes were provided by Dr. Susan Uprichard and were cultured at 37 °C with 5% CO_2_.^34^

### Cell viability assays

Cells were initially plated at a density of 5×10^5^ cells per well in six-well plates the day before treatment. They were subsequently treated with either 10 mM 2DG, 50 ng/ml TRAIL or both for 24 h unless otherwise indicated. Viability was assessed with PE-conjugated antibody against activated caspase-3 according to the manufacturer’s protocol (BD Pharminogen, San Jose, CA, USA; 550914). Cells were fixed with Cytofix/Cytoperm solution on ice for 20 min, before being stained with the antibody at room temperature for thirty minutes. Samples were washed before performing flow cytometry. Alternatively, cell death was detected by Annexin V/PI double staining (BioVision, Milpitas, CA, USA; K101-25). Briefly, cells were resuspended in 1× Binding Buffer before adding equal parts Annexin V-FITC and PI. Samples were incubated at room temperature for approximately 5 min before performing flow cytometry. Finally, TMRM was used to assess mitochondrial depolarization. Cells were stained with 100 *μ*M TMRM for 30 min at 37 °C, before washing and performing flow cytometry.

### Western blotting

PVDF membranes were blocked in Tris-buffered saline and 0.1% Tween-20 (TBST) with 3% BSA and 5% skim milk overnight at 4 °C. Primary antibodies used in western blotting were prepared in a solution of TBST with 5% BSA at a working concentration of 1 : 1000 unless recommended otherwise by the manufacturer and included antibodies against Bid (2002), BiP (3177), Caspase-3 (9665), Caspase-8 (4790), Caspase-9 (9502), COX IV-HRP Conjugate (5247), Death Receptor 5 (8074), anti-MEK1/2 (9126), anti-ERK1/2 (137F5) (4695), anti-phosph-MEK1/2 (Ser217/221) (9154) and anti-phospho-ERK1/2 (Thr202/Tyr204) (4376) all from Cell Signaling Technology (Danvers, MA, USA); Death Receptor 4 (ab8414; AbCam, Cambridge, MA, USA); Anti-*β*-actin (A4700; Sigma); and HRP-conjugated goat anti-rabbit IgG (sc-2020; Santa Cruz Biotechnology, Santa Cruz, CA, USA). HRP-conjugated goat anti-rabbit IgG secondary antibody (Promega, Madison, WI, USA; SA1-9510) was used at a concentration of 1 : 5000 and detected using an enhanced chemiluminescence detection system (Amersham, Pittsburgh, PA, USA; RPN2132, RPN2133). Semiquantitative densitometry was performed using NIH Image J software (ImageJ, US National Institutes of Health, Bethesda, MD, USA).

### Abrogation of apoptosis

General caspase inhibitor, Q-VD-OPh, was reconstituted in DMSO (R&D Systems, Minneapolis, MN, USA; OPH001). When used in apoptosis assays, the inhibitor was supplemented to the media at a concentration of 20 *μ*M. 5×10^5^ HT-29 cells were transfected with 1 *μ*g of either the CrmA or DN-FADD plasmid constructs using Effectene (Qiagen, Valencia, CA, USA; 301425). Stable cell lines were generated by selecting with 2 mg/ml Gentamicin (Life Technologies, Carlsbad, CA, USA; 15710-072) for approximately 2 weeks. Gentamicin-resistant cells were maintained under selection pressure until 24 h before treatment.

### Cell fractionation

Cell fractionation and enrichment of the heavy membrane and cytoplasmic fractions were achieved according to the AbCam subcellular fractionation protocol. Cell fractionation and enrichment of the heavy membrane and cytoplasmic fractions were achieved following the subcellular fractionation protocol. The subcellular fractionation buffer used consists of 250 mM sucrose, 20 mM HEPES, 10 mM KCl, 1.5 mM MgCl_2_, 1 mM EDTA and 1 mM EGTA. The buffer was supplemented with 1 M DTT and 50× protease inhibitor cocktail before use. 1×10^6^ cells were resuspended in 50 *μ*l fractionation buffer and incubated on ice for 20 min to allow for lysis. Lysates were centrifuged at 3000 r.p.m. for 5 min to precipitate the nuclear fraction, which was discarded. The supernatant was centrifuged again at 8000 r.p.m. to pellet the mitochondrial fraction. The supernatant was used as the cytosolic fraction while the mitochondrial pellet was dispersed by passing it through a 25G needle approximately 10 times and centrifuged again at 3000 r.p.m. The supernatant was subsequently removed and sonicated briefly on ice at a power of 2-continuous.

### RNA isolation and cDNA synthesis

RNA was extracted using Trizol (Molecular Research; 15596-018). Cells were homogenized in 1 ml Trizol for every 5×10^6^ cells. Added 0.2 ml chloroform, vortexed and centrifuged at 13000 r.p.m. at 4 °C. The aqueous phase was collected and mixed with 0.5 ml isopropyl alcohol and incubated at room temperature before centrifuging at 13 000 r.p.m. Isopropyl alcohol was removed completely and the RNA pellet was resuspended in 1 ml 75% ethanol and vortexed. RNA was pelleted again at 13 000 r.p.m. and allowed to dry before resuspending in RNAse-free water. For synthesis of cDNA for QRT-PCR of DR expression levels, SuperScript III First-Strand Synthesis System was utilized according to the manufacturer’s protocol (Invitrogen, Grand Island, NY, USA; 18080-051). RNA was mixed with OligoDT and incubated in a thermocycler for 5 min at 65 °C followed by 1 min at 25 °C. The mix was then supplemented with DTT, 5× buffer, reverse transcriptase and dNTP. This was followed by incubation at 50 °C for 1 h. After adding TE buffer the reaction was incubated at 70 °C for 15 min.

### Cell surface expression of DRs

Surface expression of DR4 and DR5 was assessed by flow cytometry after suspension in biotin-conjugated DR4 (eBioscience; 13-6644), DR5 (eBioscience; 13-9908) or Mouse IgG1 K Isotype Control antibody followed by staining with streptavidin PE (eBioscience; 12-4317-87).

### Quantitative real-time PCR (QRT-PCR) of DRs

DR4 mRNA levels were assessed by qPCR using the forward primer 5′- CTG AGC AAC GCA GAC TCG CTG TCC AC-3′ and the reverse primer 5′- AAG GAC ACG GCA GAG CCT GTG CCA T-3′. DR5 mRNA levels were assessed by qPCR using the forward primer 5′- CTG AAA GGC ATC TGC TCA GGT G-3′ and the reverse primer 5′- CAG AGT CTG CAT TAC CTT CTA G-3′. DR transcript levels were normalized to the internal control, GAPDH using the forward primer 5′- CTC CCC ACA CAC ATG CAC TTA-3′ and the reverse primer 5′- CCT AGT CCC AGG GCT TTG ATT-3′. QRT-PCR was conducted using SYBR Green (Life Technologies; 4309155). Normalization was calculated as follows: ΔC_T _(normalized gene expression)=C_T _(death receptor 4/5)−C_T_(GAPDH). Relative expression was then calculated using the formula: 2^−ΔΔCT^ where ΔΔC_T_=ΔC_T_ (normalized gene expression with 2DG treatment)−ΔC_T _(normalized gene expression without 2DG) and expressed as arbitrary units.

### Lentiviral vectors and transduction

The lentiviral construct used for shRNA delivery was derived from the pLKO.1 TRC cloning vector (Addgene, Cambridge, MA, USA; 10878). Oligos were designed using validated sequences for TNFRSF10B (DR5) (Sigma-Aldrich). The sequences for DR5 shRNA-1 and -2 were derived from clones SHCLNG-NM_005932 and SHCLNG-NM_005933 respectively. Oligo design, annealing, restriction digestion and ligation of annealed oligos into the pLKO.1 vector were performed following the manufacturer’s protocol. Lentiviral particles were produced by transfecting by the calcium phosphate method into 293 T cells with 1 *μ*g pLKO.1 shRNA plasmid, 750 ng psPAX2 packaging plasmid (Addgene; 12260) and 750 ng pMD2.G envelope plasmid (Addgene; 12259). Cells were cultured for 24 h before harvesting the supernatant on two consecutive days. Subsequently, 5×10^5^ HT-29 were plated in six-well plates. Twenty-four  hours after plating, media was removed and lentiviral supernatant was added to each well, covering the cells. This was incubated at 37 °C for 6 h before supplementing with 1 ml DMEM. Media was changed 24 h later. Cells successfully transduced were selected for using puromycin (Sigma; P8833).

### Nanostring microRNA analysis and kinase inhibitor assay

Total RNA was isolated from HT-29 cells after 18 h treatment with 2DG, TRAIL or both using the standardized Trizol protocol described above. MicroRNA was enriched using the miRNeasy Mini Kit (Qiagen; 217004). RNA samples were evaluated as biological replicates ensuring that the RNA concentration was 100 ng/*μ*l with a 260/280 greater than 1.9 and a 260/230 greater than 1.8. Analysis was performed using the NanoString nCounter gene expression system.^34^ Normalization of the raw data was performed by NanoString. The geometric mean of the top 100 expressed miRs was calculated for each assay. Using these means, a normalization factor was calculated for each assay to account for miRNA sample content variation. Subsequent replicate analysis demonstrated reproducibility between samples with *R*^2^ values ranging from 0.92 to 0.98. The nCounter assay also included a set of six internal, positive control probes and eight negative control probes. The highest count detected from a negative control was 24, which was used as the limit of reliable detection of other miRs in the assay.

For kinase inhibitor screening, 8×10^3^ HT-29 cells were seeded and treated with 10 mM 2DG, 50 ng/ml TRAIL and compounds from the kinase inhibitor library (Enzo Life Sciences, Farmingdale, NY, USA; BML-2832).

### MicroRNA inhibitors and mimics, plasmids

MicroRNA inhibitors were purchased from Exiqon (Woburn, MA, USA) including miRCURY LNA Power Inhibitor Control (199020-04), miRCURY LNA Power Inhibitor hsa-miR-494 (427173-04), miRCURY LNA Power Inhibitor has-miR-1246 (426697-04) and a custom miRCURY LNA Power Inhibitor hsa-miR-4516. MicroRNA mimics were purchased from Qiagen, including Syn-hsa-miR-494 (MSY0002816), Syn-hsa-miR-1246 (MSY0005898) and Syn-hsa-miR-4516 (MSY0019053). CA-MEK1, dn-MEK1, and dn-ERK1 and dn-ERK2 plasmids were kindly provided by Dr. Sekhar Reddy. Plasmid DNA purification kit, miRNeasy mini kit (217004) and miScript II RT kit (218160; Qiagen) were used according to the manufacturer's protocols. Transfection was performed by electroporation using the Amaxa Cell Line Nucleofector Kit R (Lonza; VCA-1001) and transfection efficiencies confirmed. Gene expression, activation and apoptosis analysis were performed 24 h after electroporation.

### FACS analysis

Prior to analysis, cells were harvested using enzyme-free dissociation buffer (Invitrogen) and washed once with PBS prior to being stained as described in detail above. Samples were washed once again with PBS prior to analysis. Where indicated only GFP-positive cells were analyzed. FACS was performed using a FACSCalibur (BD Biosciences).

### QRT-PCR of microRNA

Upregulation of four of the microRNAs found in the NanoString analysis were confirmed by QRT-PCR. Validation of hsa-miR-494, -1246 and -4516 was conducted using miScript Primer Assays (MSY0002816, MSY0005898 and MSY0019053, respectively). cDNA template for these assays was generated using the miScript II RT Kit (Qiagen; 218160), QRT-PCR was performed using the miScript SYBR Green PCR Kit (Qiagen; 218073) and signal was normalized to RNU6B levels (Qiagen; MS00033740). Primers for hsa-miR-4488 were purchased from Applied Biosystems (Foster City, CA, USA) (AM17100). cDNA template for this assay was generated using TaqMan MicroRNA Reverse Transcription Kit (Applied Biosystems; PN4366596), QRT-PCR was performed using the TaqMan 2X Universal PCR Master Mix (Applied Biosystems; PN4324018) and signal was normalized to RNU6-2 levels (Applied Biosystems; AM001093).

### *In vivo* studies

Eight-week-old athymic nude mice weighing approximately 20 g (Charles River Laboratories; Strain 490) were subcutaneously injected with 5×10^6^ HT-29 (GFP-Luc^+^) cells. One week was allowed for palpable tumor formation at which point treatment was initiated. Mice were treated for five consecutive days as in human clinical trials. 2DG was administered i.p. at 0.5 mg/kg in the morning. TRAIL (100 *μ*g/10 mm^3^ tumor volume) or control diluent was injected intralesionally 12 h after 2DG administration. Tumor size was monitored by direct measurement using digital calipers. Volume was calculated by *V*=4/3 *π* (*r*_1_)(*r*_2_)(*r*_3_). Mouse xenografts were also assessed by *in vivo* imaging using the Xenogen IVIS Spectrum from Caliper Life Sciences (Waltham, MA, USA). D-Luciferin (Caliper LIfe Sciences; 122796) was prepared as a 15 mg/ml stock solution in PBS and filter sterilized through a 0.2 *μ*m filter. Mice were injected i.p. with 10 *μ*l/g of body weight. Images were acquired using Living Image software (Caliper LIfe Sciences).

### Statistical analysis

Results are expressed as means±S.E.M. Comparisons between means were evaluated by Student’s *t*-test. A one-way analysis of variance was used for multiple comparisons. A value of *P*<0.05 was considered significant.

## Abbreviations

2DG: 2-deoxy-D-glucose
DISC: death-inducing signaling complex
DR: Death Receptor
FDG: 2-deoxy-2-fluoro-D-glucose
miRNA or miR: microRNA
TRAIL: tumor necrosis factor-related apoptosis-inducing ligand.
