# Supplementary figures and images for: Targeting the metabolic pathway of human colon cancer overcomes resistance to TRAIL-induced apoptosis
Source: Cell Death Discov. 2016 Sep 12;2:16067–. doi: 10.1038/cddiscovery.2016.67 (PMC5018545; doi:10.1038/cddiscovery.2016.67)

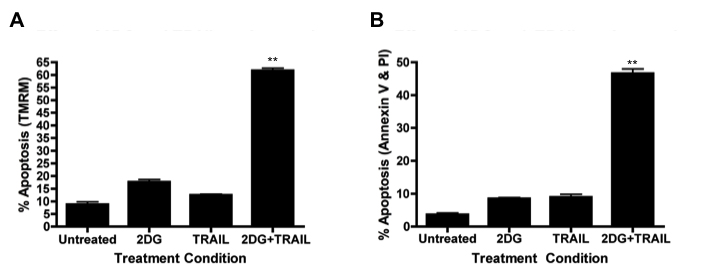

Supplement: Supplementary Figure 1 [file cddiscovery201667-s1.jpg]

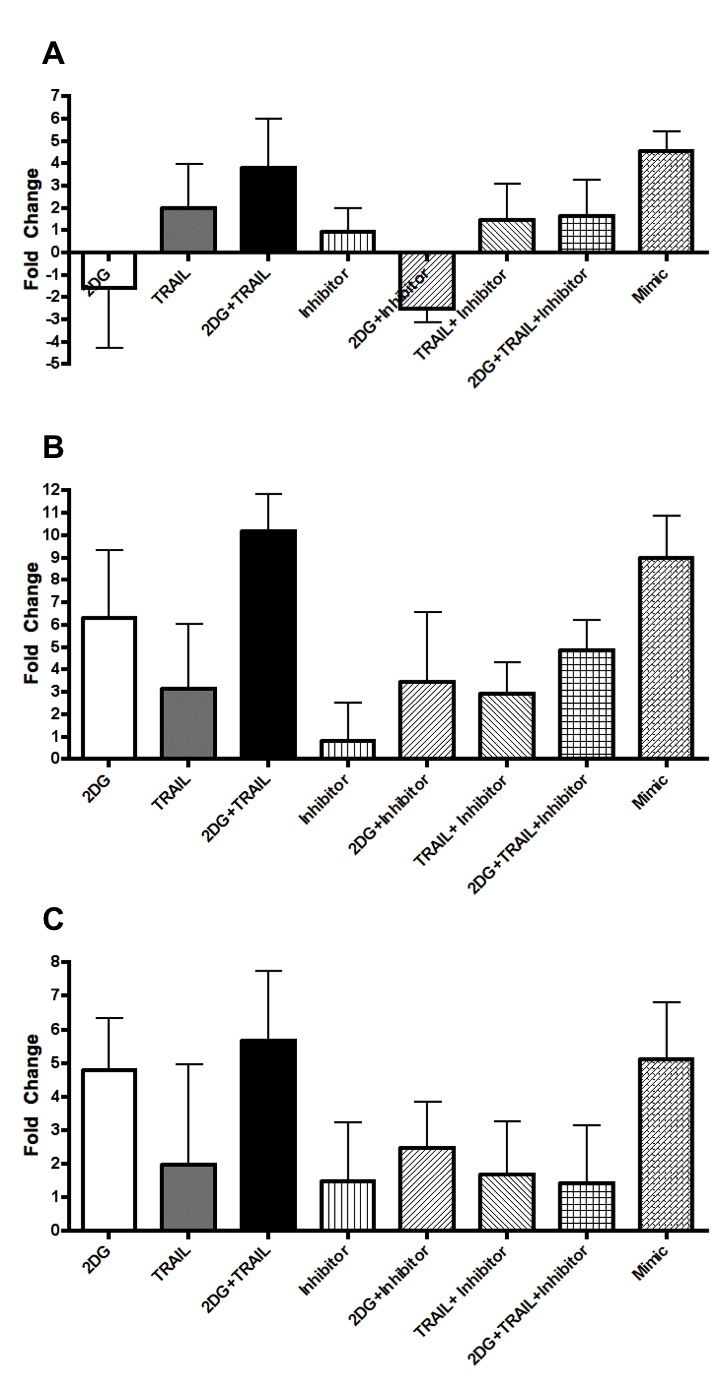

Supplement: Supplementary Figure 2 [file cddiscovery201667-s2.jpg]
