# Supplementary material for: Targeting the metabolic pathway of human colon cancer overcomes resistance to TRAIL-induced apoptosis
Source: Cell Death Discov. 2016 Sep 12;2:16067–. doi: 10.1038/cddiscovery.2016.67 (PMC5018545; doi:10.1038/cddiscovery.2016.67)
Supplement: Supplementary Information [file cddiscovery201667-s3.pdf]

Supplemental Data

Table I. SIGNIFICANTLY REGULATED miRs

Fold Change > 1.50 or < 0.66 Relative to Control

miR	*2DG*	*TRAIL*	*2DG+TRAIL*
let-7a-5p	**1.57**	1.11	1.44
let-7e-5p	**1.61**	1.02	1.35
let-7g-5p	**1.67**	1.22	1.48
7-5p	1.18	**1.51**	1.46
21-5p	**1.94**	1.45	**1.79**
22-3p	**1.60**	1.19	**1.96**
222-3p	1.18	**1.58**	**1.55**
23a-3p	1.12	**1.59**	**1.62**
26b-5p	**1.55**	1.27	**1.77**
29b-3p	**1.85**	1.49	**1.69**
29c-3p	1.45	1.27	**1.55**
30d-5p	1.24	**1.62**	**1.65**
33a-5p	**1.59**	1.49	0.99
96-5p	**1.95**	1.06	1.33
99b-5p	**1.62**	1.24	1.44
125a-5p	**1.64**	1.19	1.48
132-3p	**1.59**	1.44	**1.66**
148a-3p	**1.89**	1.03	**2.10**
151a-5p	**1.79**	1.30	1.23
192-5p	**1.51**	1.25	1.29
194-5p	1.15	1.36	**1.51**
200c-3p	1.50	1.17	**1.63**
215	0.90	**0.64**	1.00
222-3p	1.18	**1.58**	**1.55**
301a-3p	1.39	1.35	**1.59**
320e	1.41	1.24	**1.96**
361-5p	**1.60**	1.28	**1.64**
365a-3p	**2.10**	1.03	**1.84**
454-3p	1.36	**1.64**	**1.87**
**494**	1.02	**1.87**	**4.63**
574-3p	**1.89**	1.11	**1.63**
574-5p	**1.86**	1.29	**2.03**
582-5p	**1.86**	1.29	**2.03**
598	**1.55**	1.40	1.40
**1246**	0.80	**1290.08**	**2313.15**
1247-5p	**1.63**	**3.61**	**3.04**
3676-3p	0.69	1.02	**0.54**
4284	0.72	**1.87**	**2.33**
4286	1.18	**1.86**	1.49
4485	0.99	**9.83**	**8.15**
**4488**	**1.58**	**4.18**	**23.66**
**4516**	1.09	1.28	**3.75**

Blue is indicative of a fold change > 1.50 relative to control levels

Red is indicative of a fold change < 0.66 relative to control levels

Table II. EFFECT OF MAP KINASE SIGNALING INHIBITOR ON 2DG+TRAIL

Compound	*Target*	*2.7 µM*	*8.3 µM*	*25 µM*
**PD98059**	MEK	+	+	+++
**U-0126**	MEK	+	++	+++
**SB-203580**	p38 MAPK	-	+	+++
**SB-202190**	p38 MAPK	-	+	--

+++ indicates apoptosis comparable to untreated controls
++ indicates apoptosis < TRAIL but > untreated controls
+ indicates apoptosis < 2DG+TRAIL but > TRAIL
- indicates apoptosis comparable to 2DG+TRAIL
-- indicates apoptosis > 2DG+TRAIL
